# Substance Use Disorders as a Critical Element for Decision-Making in Forensic Assertive Community Treatment: A Systematic Review

**DOI:** 10.3389/fpsyt.2021.777141

**Published:** 2021-12-07

**Authors:** Thomas Marquant, Meike Van Nuffel, Bernard Sabbe, Kris Goethals

**Affiliations:** ^1^Department of Psychiatry, Collaborative Antwerp Psychiatric Research Institute, Antwerp, Belgium; ^2^Department of Forensic Psychiatry, Fivoor, Rotterdam, Netherlands; ^3^Department of Justice, Brussels, Belgium; ^4^Department of Psychiatry, VZW Walden, Leuven, Belgium; ^5^Department of Psychiatry, Antwerp University, Antwerp, Belgium; ^6^Department of Psychiatry, Antwerp University Hospital, Antwerp, Belgium; ^7^Department of Forensic Psychiatry, University Forensic Center, Antwerp, Belgium

**Keywords:** substance use disorders, forensic assertive community treatment, addiction, forensic rehabilitation, mentally ill offenders

## Abstract

**Introduction:** The prevalence of substance use disorders in forensic populations is high. They are an important factor linked to negative outcomes in mentally ill offenders and are detrimental to forensic or non-forensic outcome measures. In contrast, substance use disorders are often underdiagnosed and undertreated, especially in forensic settings. Forensic Assertive Community Treatment is a forensic adaptation of regular assertive community treatment, combined with essential elements of forensic rehabilitation theories. Little is known however on the effectivity of forensic assertive community treatment when it comes to substance use disorders or what their exact role is on the outcome measures. In this paper, we explore how SUD is treated in Forensic assertive community treatment and how it relates to the forensic and non-forensic outcome measures.

**Methods:** We performed a systematic review (PRISMA) of forensic Assertive community treatment teams that followed the main evidence-based principles of regular assertive community treatment and added basic elements of forensic rehabilitation. We analyzed articles the Psychinfo and Medline databases dating from 2005 to 2020. Fifteen studies fit the search criteria and were included in the analysis. The Quality of the studies was assessed using the Newcastle-Ottawa scale.

**Results:** SUD was highly prevalent in all studies. Patients entered FACT through two pathways, either from a care continuum or directly from prison. The severity of SUD at intake emerges as a critical element when deciding which pathway to choose, as a high severity-score at the start of FACT follow-up was linked to recidivism. While differing in method all studies offered integrated SUD treatment. These included evidence-based techniques like CBT, therapeutic communities, and Substance Abuse Management Module. Though results on SUD outcomes were mixed 4 studies mentioned abstinence in 50–75%. The severity of SUD tended to increase initially and to stabilize afterwards.

**Conclusion:** Severity of SUD at intake emerges as a decisive element in decision-making on entering FACT teams directly from prison or through a care-continuum. The ways to provide SUD treatment varied and outcomes for SUD were mixed. SUD was found to be detrimental to forensic and non-forensic outcome measures, such as recidivism or hospitalizations during FACT treatment.

## Introduction

In all forensic settings, offenders with mental illness are known to have high rates of substance use disorders (SUDs) (1–7). SUDs are more prevalent in forensic populations than in the non-forensic population or the general population, and having a SUD is a known risk factor for patients that leads to entering forensic services (8–11). The prevalence of SUDs is also increasing within forensic populations (12, 13). Furthermore, SUDs are linked to violent and non-violent recidivism (12–25). Additionally, SUDs are linked to other adverse outcomes, such as death, absconding, injury, escapes, and rehospitalization (3, 7, 26–30). The latter is especially prevalent in combination with antisocial personality traits and impulsivity. The presence of SUDs can also predict violent offending and reoffending (31–34) and are linked to antisocial traits and impulsivity (11). Violent offending and SUDs often go hand-in-hand as violent offenders are often intoxicated or under the influence of substances at the time of the offense (21, 35, 36). Research has also shown that SUDs often remain undertreated, worsening the prognosis of mental health disorders and leading to avoidance of care (37–39). The presence of a SUD is also an indicator or predictor for mental health disorders (40–44).

Besides suffering from the detrimental consequences of SUDs, forensic patients with SUDs also have low responsivity toward desistance programs, especially regarding increasing of motivation to stop or reduce substance use (44–46). In their study, Delaney reported that up to 83% of patients continued to have a SUD, and in Clausen et al.'s study (47), this was 93% (44). Targeting the treatment of SUDs requires flexibility and innovation from organizations (48). SUDs are increasingly regarded as chronic disorders, requiring chronic follow-up (49). Substance use is a known risk in psychotic disorders, as it can increase the likelihood of violent behavior (14).

Unfortunately, evidence on what works in treating forensic patients with SUDs is limited, either in residential or community-based settings (50). A Cochrane database review from 2015 showed that the therapeutic communities' intervention had a significant statistical effect (51). This finding was supported by Sacks et al. (52), who adapted the therapeutic community in a re-entry program following incarceration. For mentally ill patients, the Cochrane review mentioned a cognitive behavioral curriculum, psychoeducation, and the heightening of treatment engagement as effective, but not statistically significant. According to Marlowe (53), identified community-based programs, close supervision, certain and immediate consequences, and diversion are essential elements of successful programs for treating SUDs.

Assertive community treatment (ACT) is a well-known approach to deliver community-based psychiatric follow-up for patients suffering from serious mental illness (54). ACT was developed as an alternative to hospitalization for patients with serious mental illness and relies on a multidisciplinary team providing intensive contact through home visits. A large body of literature provides evidence in support of the effectiveness of ACT regarding non-forensic outcome measures, such as the number of hospital admissions, length of stay during hospital admission, quality of life, adherence to treatment, clinical outcome, and patient satisfaction (55–61). Including treatment for substance use in ACT is considered essential for the outcome (62–65).

Penzenstadler et al. (66) reviewed the effectiveness of non-forensic ACT of SUD outcomes regarding housing, substance use, treatment engagement, legal problems, and hospitalization rates. The study used 11 randomized controlled trials (RCTs) with positive results for hospitalization rates and treatment engagement. The study observed that higher fidelity to the ACT model improved outcomes. Substance use was reduced in half of the studies, but only one study favored ACT for treating substance use. There was no reduction in criminal behavior in the ACT group (67), but patients were less likely to end up in jail (68). Staff working in regular settings struggled to engage patients with antisocial personality traits or disorders, which may have been detrimental to the outcomes (69). These poor effects on forensic outcome measures such as jail time or arrests are in accordance with prior research indicating a lack of effect on forensic outcome measures for non-forensic ACT (65, 70, 71). Overall, the review concluded that the results varied significantly (66). Nevertheless, ACT was considered to be a promising way to deliver psychiatric care to patients suffering from SUDs. In all studies, methodological limitations were an issue. A large study in the Netherlands using ACT did see a reduction in SUD-related problems during the follow-up period, resulting in less SUD-related admissions (61).

Forensic ACT (FACT) can be conceptualized by adapting regular ACT so that it retains the evidence-based elements (62) toward clinical outcomes, while incorporating essential aspects of forensic psychiatric care (72–75). The effectiveness of FACT on forensic outcome measures has been established in previous studies (76–81). For FACT teams to work effectively, they need to offer round-the-clock service, integrated SUD treatment, low caseloads, and provide patient contact through home visits, an embedded psychiatrist, and vocational services. Additionally, FACT teams need to apply the hybrid functioning of a clinician, combining therapeutic tasks with control tasks. This is demonstrated by working closely with justice departments as a form of leverage (75) and conducting formal risk assessment during intake and follow-up (80).

In this current review, we aim to assess how effective FACT is for treating SUDs and how SUDs are related to forensic and non-forensic outcomes. Therefore, the following research questions were investigated:

1. How are substance use disorders treated in forensic assertive community treatment?
2. How effective is forensic assertive community treatment for substance use disorders?
3. How do substance use disorders influence forensic and non-forensic outcome measures?

## Methods

**Figure 1:**
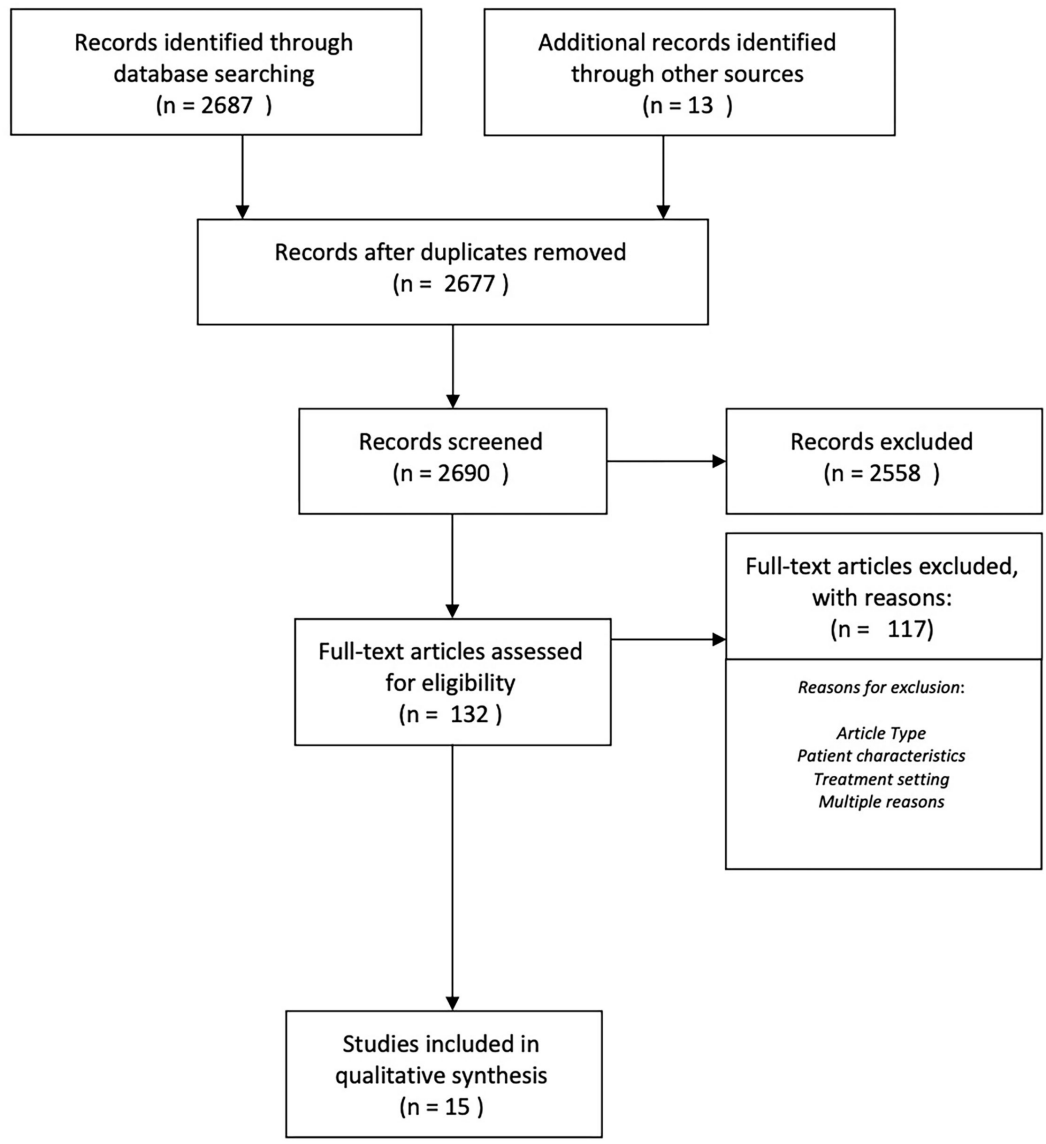
PRISMA flow diagram.

To investigate the research questions stated above, we conducted a systematic review using the PRISMA methodology on studies conducted between 2005 and 2020 (82). A PRISMA flow diagram is added in Figure 1. We searched PubMed and PsycINFO with the following search criteria: “*forensic psychiatry* + *community care* + *substance use* + *treatment*”, “*assertive community treatment* + *substance use*”, “*substance use* + *treatment* + *forensic psychiatry*”, “*drug treatment program* + *forensic psychiatry*”. For an overview of the search results, please consult Figure 1. The search results yielded a total of 2,687 hits and an additional 12 hits were added after screening the references of relevant reviews. One study was added after receiving a study ahead of print, which was published later on (75). After removing duplicates, 2,677 studies remained. In total, 2,690 records were screened by title, for which the screening criteria were as follows: *forensic, (assertive) community (treatment), case management, and/or substance (ab)use*. After the screening process, 132 full articles were read. At this point, we excluded articles for reasons related to the article type. As such, we excluded reviews (15), book chapters (1), study protocols (8), conference texts (1), dissertations (2), studies on policy implementation (1), and comments (1). Next, we excluded studies based mainly on patient characteristics. These were studies with a focus on a primary diagnosis of intellectual disability (1), studies focusing on posttraumatic stress disorder (PTSD) (1), studies where patients had no SUDs (2), studies that did not require the included population to have a mental illness (8), or studies on patients that were not referred through the justice system (23). Then, we excluded studies based mainly on the treatment setting. These were studies conducted in residential care (5), studies on Housing First (2), or studies on outpatient clinics without outreach (3), Lastly, we excluded studies that were irrelevant for multiple reasons (such as the abovementioned) (34).

Out of the 24 remaining studies, only 15 were identified to work with a FACT team. To identify which studies worked with such teams, we screened for the six evidence-based elements of regular ACT and the two forensic elements (72, 73). Studies needed to offer integrated treatment for SUDs, an embedded psychiatrist, around-the-clock service, low caseloads, and vocational services. Additionally, the teams needed to work closely with justice services and apply a hybrid stance toward patients, combining treatment and risk assessment (80). To be included in this review, the two forensic elements was mandatory needed to be present, as well as the six evidence-based elements of regular ACT. Nine studies offered services to forensic patients with SUDs, but they did not have the two forensic elements required and, as such, were identified as studies with regular ACT (8, 61, 67, 68, 83–86). As stated before, we were left with 15 studies that could be included in the qualitative analysis, reporting on nine datasets. Two studies were combined into one, because one study (73) described the model, while the second study reported on the outcomes (87). As such, our review includes 14 studies.

The quality of the studies was assessed using the Newcastle-Ottawa scale (NOS), which is commonly used to assess the quality of case–control studies and cohort studies (88). For time at risk, we used a minimum of 1 year follow-up, based on the fact that 12 months was a critical point in earlier studies: at this point, 50% of abstinent patients remained abstinent for another year (53).

## Results

### Overview of Studies Included

An overview of the studies is presented in Table 1, along with the main characteristics, such as number of patients, presence of a control group, time at risk, follow-up, primary diagnosis, diagnostic information on the presence of SUDs, SUD treatment information present, SUD outcomes present, and score on the NOS. Twelve studies were conducted in the US, one study was conducted in Belgium (80) and one in New Zealand (93). The sample size ranged from 8 to 137 patients. The time at risk ranged from 270 to 1,274 days, with an average of 531 days.

**Table 1:** Overview of included studies.

**References**	***N***	**Control group**	**Time at risk (days)**	**Primary diagnosis**	**SUD diagnosis prevalence (%)**	**Reported on SUD treatment**	**Reported on SUD outcomes**	**Newcastle-Ottawa score**
Solomon and Draine (89)	60	RCT	365	SMI	NS	No	Yes	4
Lurigio et al. (90)	8	No	NS	SMI	Highly present	NS	No	2
Cimino and Jennings (91)	18	No	508	Psychosis/Bipolar	100	Yes	Yes	2
Parker (76)	40	Yes	1,274	SMI/Psychosis	42	Yes	No	5
McCoy et al. (92)	24	No	730	SMI	Highly present	Yes	Yes	2
Simpson et al. (93)	105	No	660	SMI/Psychosis	78	Yes	Yes	5
Cosden et al. (94)	137	RCT	540	SMI	83	Yes	Yes	6
Davis et al. (95)	96	No	365–1,095	SMI/Axis-1	Present	NS	No	2
Erickson et al. (87)	130	No	882	Psychosis	67	Yes	Yes	5
Smith et al. (78)	91	No	495	Psychosis/Bipolar	100	Yes	Yes	4
Cusack et al. (77)	72	RCT	365–730	SMI/Axis-1	66	Yes	No	7
Kelly et al. (96)	22	No	270	SMI	55	Yes	Yes	2
Lamberti et al. (79)	35	RCT	329	Psychosis	70	Yes	Yes	6
Marquant et al. (80)	70	Yes	663	Psychosis/Personality dis.	81	Yes	Yes	6

*NS, Not Specified; RCT, Randomized Controlled Trial; SMI, Serious Mental illness*.

We found several articles reporting on the same projects at different stages. Lamberti et al. (73, 79) and Erickson et al. (87) reported on a project in Rochester, New York. Lurigio et al. (90), McCoy et al. (92), Davis et al. (95), and Kelly et al. (96) reported on the Thresholds program in Chicago. Smith et al. (78) and Cimino and Jennings (91) reported on the Arkansas Partnership Program. The design of the studies all included the evidence-based elements of regular ACT and the two forensic elements (72, 75). Five studies reported on a continuum of care where patients went through a residential setting before being treated by a forensic FACT team (76, 78, 80, 91, 93). The other articles included studies on patients who had been incarcerated.

Between the different articles, study design varied. Only four studies used RCTs (74, 77, 89, 97), while only one study used non-randomized controlled design (80). One study had no control group, but it compared outcomes by splitting the study group into two (76). The study by Cosden et al. (97) combined a mental health court with FACT. Similarly, Lamberti et al. (79) described a FACT team working closely with a judge in weekly meetings. Two studies were identified to be qualitative and/or descriptive (90, 92). The studies had different exclusion criteria (i.e., assessment at intake) for excluding certain patient groups, such as high-risk patients or violent offenders. In the studies that reported on a continuum of care, the exclusion criteria were less restrictive and patients with violent offenses were not excluded.

In all studies, the diagnostic information for inclusion was major mental illness, mostly psychotic disorders or bipolar disorders. SUD was mentioned as a highly present comorbid diagnosis in 13 studies with an average occurrence of 74%. Only one study reports on personality disorder as the primary diagnosis in 50% of included cases (72). No studies reported on SUD as the primary diagnosis. Twelve studies mention the implementation of SUD treatment for patients and 10 report on SUD as an outcome measure.

### Quality of Analysis

The quality of the studies was assessed using the NOS for non-randomized studies in meta-analysis. TM and KG assessed the quality independently and reached a consensus in case of conflict. The scale was divided into three domains: selection (representativeness of groups, ascertainment of exposure, and outcome of interest), comparability, and outcome (assessment of outcome, time at risk, and adequacy of follow-up). To determine the quality, we followed the guidelines of the NOS by awarding stars in each of these three domains (98). A good quality score required three or four stars in selection, one or two stars in comparability, and two or three stars in outcomes. A fair score required two stars in selection, one or two stars in comparability, and two or three stars in outcomes. A lower number of stars in each domain was awarded a low score.

The results of the quality assessment are as follows: three studies achieved the status “good quality” (77, 79, 97), three achieved “fair quality” (76, 80, 93), and the rest were deemed to be “low quality.” In total, 6 out of 14 studies were considered to be fair or good quality.

### SUD Program Design

Twelve studies reported on implementing SUD treatment for their patients. The amount of information given on the programs varies per study. Five studies reported on patients being treated by a FACT team through a continuum of care and after discharge from a psychiatric hospital. The length of stay in the residential stage was long; up to 665 days on average in the Arkansas program (91). The studies that reported on the Arkansas program described five steps during residential treatment relying on the principles of a therapeutic community. Additionally, staff received 80 h of cognitive behavioral training (CBT) for treating SUDs. The aim of these steps was to integrate the SUD treatment. From Step 3 in the program, patients had follow-up through sponsors in the community such as Alcoholics Anonymous (AA) and/or Narcotics Anonymous (NA). From this point, they were also granted supervised leave from the hospital. SUD treatment was continued during conditional release from the hospital.

Similarly, patients in the Belgian study were treated by a FACT team after a stay in a psychiatric hospital (80). The residential stay consisted of a closed ward and an open ward that patients go through subsequently. It is mentioned patients could re-enter the hospital while being in the FACT team's follow-up and could move between the closed and open wards of the hospital. Substance use is mentioned as one of the reasons to re-enter the hospital. The FACT team had a dual diagnosis treatment officer in the team, available for patients with comorbid SUD. From the studies investigating a care continuum, only the Belgian study had a control group. Of the control group, 26% received integrated SUD treatment and outcome measures were controlled for the presence of an SUD (80). Simpson et al. (93) reported on a corrective, abstinence-targeted approach toward SUDs, with urine drug screening as a way to follow-up on abstinence. Parker (76) mentioned two options for the treatment of SUD in their project. Patients could participate in an intensive outpatient program, provided by a third party, or they could participate mandated attendance for a specified number of AA meetings per week.

The four studies that included RCTs all mentioned offering integrated SUD treatment to their patients. These studies included patients directly leaving prison as opposed to patients from forensic residential care settings as discussed above. Three of the studies worked closely with justice departments, and patients had weekly contact with a judge or a mental health court (77, 79, 97). Cusack et al. (77) mentioned an integrated, team-based treatment offer, yet did not elaborate further on the content of this treatment offer. Lamberti et al. (79) used the Addiction Severity Index (ASI) (99) to measure the severity of SUDs at intake and revealed low severity of SUDs at inclusion. This was due to the fact that patients entered the program after incarceration. Cosden et al. (97) described an integrated SUD treatment, which consisted of an 8-week program designed to teach mentally ill patients how to achieve sobriety. They used the Substance Abuse Management Module (SAMM) for this, in addition to drug testing (100). Just as in Lamberti et al. (79), ASI was used to assess severity of the SUD. Solomon and Draine (89) mentioned a SUD treatment offer, yet did not go into the details of this treatment offer. The study only observed a loss of model fidelity over the course of the study. The controls for each study differed depending on the presence of SUD treatment. Lamberti et al. (79) and Cosden et al. (97) mentioned that there was no SUD treatment in the control group. In Cusack et al. (77), the control group received substance use counseling. Overall, information on SUD treatment in the control group is limited in the studies with RCTs.

In the remaining studies, Kelly et al. (96) reported that the FACT team relied on substance use counselors and used ASI scores to assess SUD severity, which were low at intake. In McCoy et al. (92), SUD treatment was also included. Davis et al. (95) mentioned the intention to implement integrated dual diagnosis treatment (IDDT), yet that has not happened at the time of the study. Erickson et al. (87) reported on the presence of an unspecified SUD treatment model (73). In the reviewed studies, it can be concluded that substance use during follow-up could lead to hospital admission or incarceration.

### SUD Outcome Measures and Relations to Forensic and Non-Forensic Outcomes

Eight studies reported SUD to be an outcome measure or to be related to forensic and non-forensic outcome measures. Within the group of studies that reported on a care-continuum, Smith et al. (78) reported that 75% of the study population achieved abstinence over the study period. This meant patients had no positive drug tests. Of the study population, 49% achieved a status called “highly successful,” which meant they were abstinent, without readmission to hospital or prison, and without being arrested. Most patients, therefore, did not relapse into substance use. The status of “overall success” was achieved when patients had no readmission to hospital or prison, and 90% of patients achieved this status. Patients with schizoaffective disorders suffered more relapse in substance use, compared to patients with other psychotic diagnoses. Smith et al. (78) then grouped the patients into five primary substance dependance categories depending on the main substance patients used. Patients with heroin and cocaine use had the lowest rates of “overall success” and suffered more rearrests. Within this group, the rearrest rate was 20%, which accounted for 60% of all rearrests within the study group, indicating the importance of heroin and cocaine use when it comes to rearrests. Additionally, this group had lower community tenure compared to the other groups. The group with mixed use of alcohol and substances had the lowest abstinence rate with 64% achieving abstinence.

Marquant et al. (80) conducted a similar study in a care continuum, albeit with a non-randomized control group. As for the forensic and non-forensic outcome measures, they did correct for substance use, antisocial personality traits, and the presence of violent offending. Within their FACT population, there was a very low incarceration rate, but a high hospitalization rate. Fifty percent of patients had at least one readmission: 70% of the time caused by a relapse in substance use. Since a relapse constituted a breach of conditional release, this could also have led to incarceration. As such, hospital admissions were a way to avoid incarceration and the average length of stay was short (12 days). Within the group of patients that were admitted more than twice, the percentage of admission caused by relapse rose to 100%. As such, substance use was also responsible for the loss of community tenure following readmissions. Within the control group, 14% of incarcerations were due to substance use. In this group, almost no one was readmitted to hospital. Furthermore, in this study, 17% of patients were admitted to a long-term stay ward, due to ongoing substance use. These patients were no longer treated by the FACT team.

Simpson et al. (93) found only one readmission due to relapse in amphetamine use. In the studies using RCTs, Cosden et al. (97) reported that patients reoffending in the study group had a high severity of SUDs at intake. The FACT was only significantly more effective on forensic outcome measures, when this group was excluded from the study. All studies with RCTs reported on FACT teams treating previously incarcerated patients as opposed to FACT teams treating patients in a care continuum.

Among the remaining studies, Kelly et al. (96) described how patients at inclusion left prison with a low severity of SUDs, based on their ASI scores. After re-entering the community, this went up significantly and seemed to stabilize afterwards. Out of 22 arrests in the study group, Kelly et al. (96) mentioned that 5 arrests were directly related to SUD and that an unspecified number were indirectly related. The latter happened when patients were arrested for committing crimes to obtain money to buy illegal substances (i.e., through prostitution). In their study group, only 4% of patients were not incarcerated or admitted to hospital and substance use was an important concern, as they reported. McCoy et al. (92) reported that after inclusion, 50% of patients achieved abstinence from alcohol and/or substances. The remaining 50% of patients did not perceive their SUD to be a problem, as it indirectly reduced criminal activities related to the substance use, such as theft to pay for substances. Surprisingly, Erickson et al. (87) reported that SUDs were not a predictor of recidivism, yet pointed out there was a lack of heterogeneity in SUDs. They mentioned a non-significant reduction in substance use in the study group.

## Discussion

At this point in time, the number of studies devoted to FACT is generally limited and suffers important qualitative limitations. Of the studies reviewed in this article, only 6 out of 14 were considered to be fair to good quality, using the NOS. Only four of these studies included RCTs (77, 79, 89, 94), of which one suffered to maintain model fidelity over the course of the study (89). Additionally, only one had a non-randomized control group (80). In comparison, a similar review of regular ACT that focused on SUD treatment effectivity found 11 studies with RCTs (66). All the studies in this review were specifically designed to investigate the effectivity of ACT on SUD and comprised a total of 741 patients. However, in our review, we found that none of the studies were aimed at researching the effects on SUDs, and that all studies investigating the effectivity of FACT, focused mostly on forensic and non-forensic outcome measures. As such, providing data on SUDs was not the core research purpose in any of the studies. Previous studies that looked at SUDs in a forensic community-based team were very rare (46).

All studies complied with the six basic elements of effectivity known from regular ACT and the two basic elements of forensic care (72, 75). However, there are still great differences in the practical approach to how patients were treated and how the teams operated (75, 81). FACT is still a relatively young form of treatment and the consensus on its effectivity is a work in progress (75, 81). Previous research, however, has shown that when following the six basic elements and the two additional forensic elements of FACT, it is effective in reducing forensic outcome measures, such as incarceration, rearrest, and bookings (72, 75, 81). That is still the core goal of any forensic community-based team and stresses the importance of model fidelity of any FACT team (17, 101). This effect was achieved regardless of the interference of SUDs or the way SUDs were treated. A similar importance of model fidelity also emerges from similar research into regular ACT (66).

### SUD Program Design

**Figure 2:**
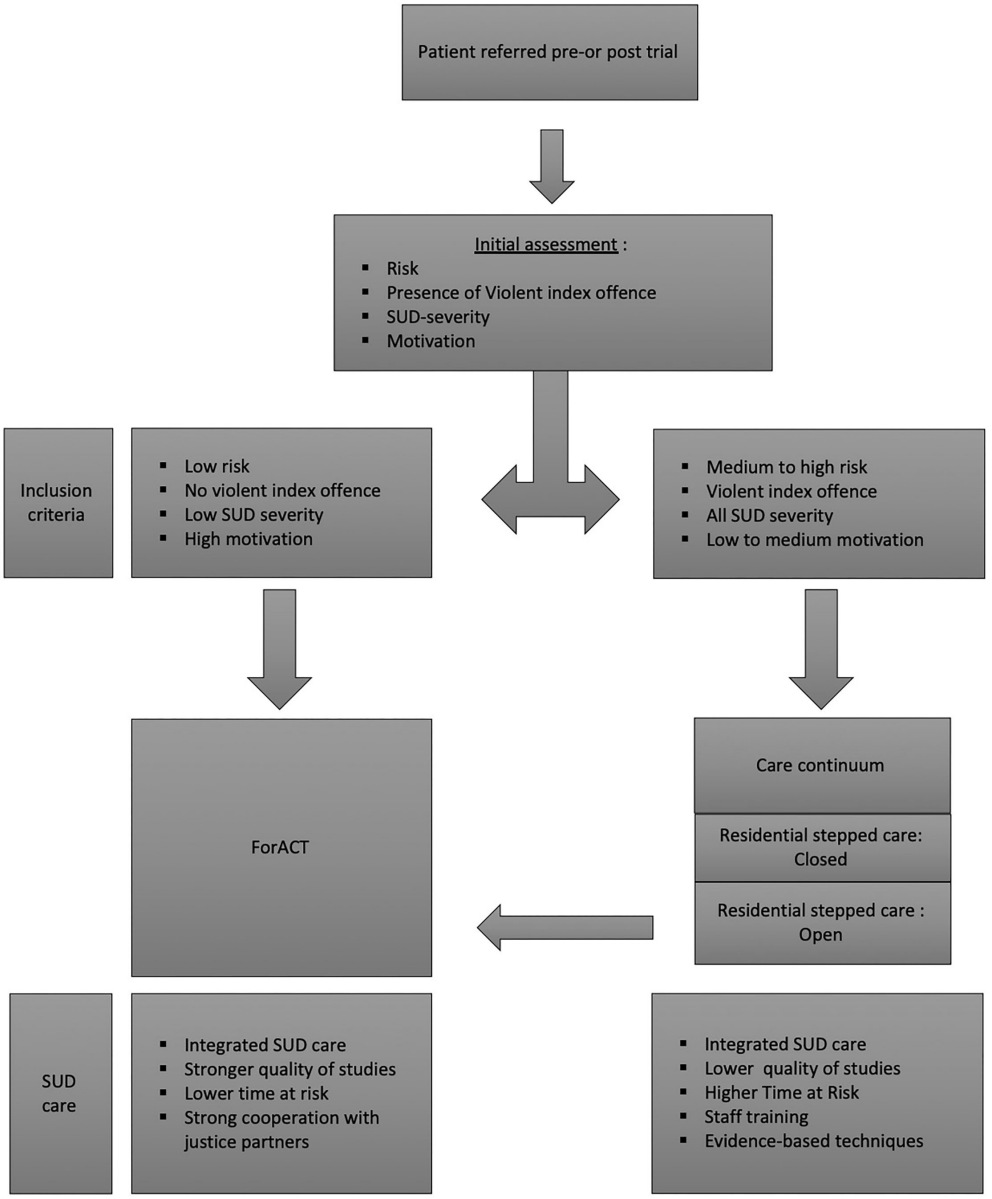
Overview of ways to deliver SUD treatment in ForACT teams.

The way the SUD treatment is delivered varied in the studies reviewed. An overview is represented in Figure 2. The initial screening was an important step in the approaches of all researched FACT teams. All teams screened for motivation and excluded patients based on the risk of or the presence of violent crimes. Several studies that also checked for the severity of SUDs at intake and reported low severity overall, except for the study done by Cosden et al. (97). Cosden et al. (97) found that a high ASI score was strongly linked to new recidivism, and to be significantly effective on forensic outcome measures, the group with high ASI scores needed to be excluded. This clearly indicates that FACT teams should assess SUDs at intake and consider that a care-continuum might be a better setting for high-risk patients (102). Recent research in the Netherlands has confirmed different risk classes in forensic patients diagnosed with SUDs (103). As a result, SUDs emerged as a critical element for decision-making on how to treat forensic patients in FACT teams and what pathway to choose.

In light of this, it is important to differentiate teams that treated previously incarcerated patients, and teams that treated patients who first went through a residential stage in a care continuum. Teams that treated previously incarcerated patients excluded high-risk patients, such as third-strikers and patients that were convicted of a violent crime (77, 79). Third-strikers are patients that receive lengthy sentences after a third subsequent crime and are, therefore, deemed to be high risk (104). These teams worked closely with justice departments, such as mental health courts, to ensure the use of leverage (75, 97). All studies with RCTs in this review that reported on previously incarcerated patients provided much stronger evidence-based results than studies that reported on patients in care continuums. Integrated SUD treatment was combined with FACT in these studies to treat previously incarcerated patients, which has been shown to be superior to non-integrated SUD treatment (64, 105). The exact nature of the treatment offers differed, and three out of four studies did not mention the use of structured community-based treatment models, such as integrated dual diagnosis treatment (IDDT). The differences make it difficult to compare the treatment approaches or to make statements on what elements contributed to effectivity. Only Cosden et al. (97) specified the use of the Substance Abuse Management Module (SAMM) as a structured community-based program (97, 100). The control groups also lacked detailed descriptions of the nature of the SUD treatments offered, yet again making comparisons difficult. This indicates that further research is needed on SUD treatment in FACT teams treating previously incarcerated patients.

The studies done in a care-continuum had less stringent inclusion criteria and did not exclude violent patients, or other high-risk patients (76, 78, 80, 93). The results from these studies were more robust because there were more data on SUDs as an outcome measure from the teams that offered FACT in a care continuum and the time at risk was longer. The care continuum FACT teams offered SUD treatments during the residential stage of the care continuum. The studies described several different approaches to treatment. Some included lengthy staff training and were based on therapeutic communities, cognitive behavioral therapy (CBT) and attending AA or NA meetings (78). Both elements of the care continuum—stepped care and therapeutic communities—are known to be effective program elements in forensic psychiatric care and SUD treatment (51, 52, 101, 106). The use of urine drug testing was frequent in all teams. One controlled study mentioned that only 26% of controls received some form of SUD treatment (80). Unfortunately, integrating FACT teams into a care continuum is expensive (107). The higher cost of treating complex forensic patients can be justified if treatment can be proven to work and as such reduce the cost of new crimes (108). Further research is needed to determine whether qualitative aftercare can reduce the length of hospital stay of patients and, subsequently, the cost of treatment. In a recent meta-analysis, which reviewed the use of psychological interventions for mentally ill people leaving prison, continuity of care emerged as an important element to successfully reduce recidivism (102).

### SUD Outcome Measures and Relations to Forensic and Non-Forensic Outcomes

Information on SUDs as an outcome measure was also reported in the reviewed studies. From the studies that reported on a care continuum, overall outcomes are good for SUDs over a long time at risk of 1 year at minimum. Both Marquant et al. (80) and Simpson et al. (93) reported that 50% of patients remained abstinent and had no readmission or rearrest and Smith et al. (78) even reported that 75% of patients remained abstinent. If patients relapsed in substance use, reincarceration and rearrest rates were still very low in both studies. However, relapses in substance use did cause a lot of hospital readmissions, but these readmissions were kept short, despite 17% of the research population ending up in long-term care for ongoing substance use (80). SUDs are a known risk factor linked to patients being transferred to long-term stay settings (109). Nevertheless, previous research has shown that new reintegration trajectories are possible and should be explored (109). Patients who are considered long-term stays are known to move a lot through the different settings of residential forensic psychiatric care (109). High-quality aftercare, such as FACT, could increase their chances of rehabilitation.

In our review, we found that SUDs interfered strongly with non-forensic outcome measures. Simpson et al. (93) found that substance use also interfered with forensic outcome measures, especially in patients using heroin and cocaine or patients combining alcohol with substances. The number of rearrests was the highest in this group. The finding that different substances resulted in different risks for recidivism has also been confirmed in a sample of not guilty for reason of insanity (NGRI) patients in the Netherlands (103). In this study, mixing alcohol and substances emerged as risk enhancers for patients with a psychotic disorder.

Although the studies with RCTs were of high quality, they gave little insight into the effects of substance use, but there were clear links between recidivism and the severity of the SUD (97). Significant results in favor of the FACT team on forensic outcome measures were only obtained after the patients with high SUD severity at intake were removed from the sample (97). This stresses the importance of a screening at intake and to include substance use severity in the decision-making on inclusion.

The remaining studies reported an increase in SUD severity at the start of follow-up and showed mixed results on treating SUDs. This is consistent with previous research stating that SUDs are a chronic state (49). These studies also reported that SUDs were strongly linked to forensic outcome measures, confirming their status as an important criminogenic factor. The studies reported similar rates of abstinence at 50% and a similar increase in SUD severity at the start of follow-up (92). However, there were mixed results on treating SUDs. What we should take away from these studies is that SUDs are strongly linked to forensic outcome measures, confirming their status as an important criminogenic factor.

### Strengths and Limitations

A strength of this review is that it is, to our knowledge, the first review dedicated to the topic of substance use in FACT, which gives it great added value to the literature on FACT. The quality of the review was ensured by using the PRISMA methodology. To conduct this review, we chose a methodology that allowed to search for literature on FACT teams that relied on the evidence-based elements of regular ACT, combined with the two essential elements of forensic rehabilitation. In this way, we were able to select studies that have model fidelity focusing on the most important forensic and non-forensic outcome measures of any FACT team. A limitation of using this methodology was that the demands for selection were very strict, and that possibly valuable studies were not included. The initial screening of the literature by title until the stage of full-text screening was done by one reviewer.

## Conclusion

FACT is a forensic adaptation of regular ACT that offers treatment to drug-using offenders affected by mental illness. We found that SUDs were highly prevalent in patients treated by FACT teams and were negatively related to all outcome measures, forensic or non-forensic. A significant number of patients did achieve abstinence. The severity of the SUD tended to increase initially and stabilized subsequently.

This review reveals that SUDs should be a decisive element in any decision-making on the risk level of patients and on the level of service intensity when referring for treatment by FACT teams. The severity of the SUD must be low at intake for previously incarcerated patients to be treated by a FACT team. Patients with severe SUDs should be treated in the residential stages of a care continuum. We found that the detrimental effects of substance use on forensic and non-forensic outcome measures highlight the need for future research on effective treatment options for SUDs in FACT to increase effectiveness. Studies on SUDs in FACT are still limited in number and quality, and caution is advised when interpreting the results of previous literature on this matter.

## Author Contributions

TM wrote the first draft of the manuscript. TM, MV, and KG contributed to conception and design of the study. All authors contributed to manuscript revision, read, and approved the submitted version.

## Conflict of Interest

The authors declare that the research was conducted in the absence of any commercial or financial relationships that could be construed as a potential conflict of interest.

## Publisher's Note

All claims expressed in this article are solely those of the authors and do not necessarily represent those of their affiliated organizations, or those of the publisher, the editors and the reviewers. Any product that may be evaluated in this article, or claim that may be made by its manufacturer, is not guaranteed or endorsed by the publisher.
